# *catena*-Poly[trimethyl­phenyl­ammonium [[bromidocad­mate(II)]-μ-bromido-μ-chlorido]]

**DOI:** 10.1107/S1600536810005817

**Published:** 2010-02-17

**Authors:** Kong Mun Lo, Seik Weng Ng

**Affiliations:** a Department of Chemistry, University of Malaya, 50603 Kuala Lumpur, Malaysia

## Abstract

In the title salt, (C_9_H_14_N)[CdBr_2_Cl], the Cd^II^ atom is five-coordinated in a trigonal-bipyramidal coordination environment. All three of the halogen sites show disorder as a result of substitution of Cl for Br or Br for Cl. Two of the three halogen atoms are involved in bridging a pair of Cd^II^ atoms, generating a linear polyanionic chain motif.

## Related literature

For the crystal structure of bis­[4-(dimethyl­amino)pyridinium]tetra­bromidocadmate monohydrate, see: Lo & Ng (2009 ▶).
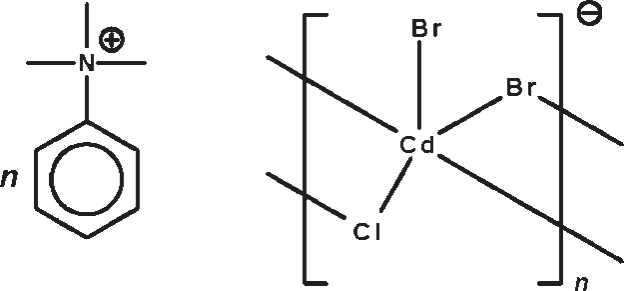


## Experimental

### 

#### Crystal data

(C_9_H_14_N)[CdBr_2_Cl]
*M* *_r_* = 443.88
Monoclinic, 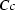

*a* = 12.9403 (2) Å
*b* = 14.7059 (2) Å
*c* = 7.3866 (1) Å
β = 95.1590 (8)°
*V* = 1399.97 (3) Å^3^
*Z* = 4
Mo *K*α radiation
μ = 7.43 mm^−1^
*T* = 293 K
0.30 × 0.25 × 0.20 mm

#### Data collection

Bruker SMART APEX diffractometer
Absorption correction: multi-scan (*SADABS*; Sheldrick, 1996 ▶) *T*_min_ = 0.378, *T*_max_ = 0.746
6431 measured reflections
3068 independent reflections
2966 reflections with *I* > 2σ(*I*)
*R*_int_ = 0.026

#### Refinement

*R*[*F* ^2^ > 2σ(*F* ^2^)] = 0.027
*wR*(*F* ^2^) = 0.070
*S* = 1.06
3068 reflections
124 parameters
10 restraints
H-atom parameters constrained
Δρ_max_ = 0.67 e Å^−3^
Δρ_min_ = −0.68 e Å^−3^
Absolute structure: Flack (1983 ▶), 1451 Friedel pairs
Flack parameter: 0.021 (9)

### 

Data collection: *APEX2* (Bruker, 2009 ▶); cell refinement: *SAINT* (Bruker, 2009 ▶); data reduction: *SAINT*; program(s) used to solve structure: *SHELXS97* (Sheldrick, 2008 ▶); program(s) used to refine structure: *SHELXL97* (Sheldrick, 2008 ▶); molecular graphics: *X-SEED* (Barbour, 2001 ▶); software used to prepare material for publication: *publCIF* (Westrip, 2010 ▶).

## Supplementary Material

Crystal structure: contains datablocks global, I. DOI: 10.1107/S1600536810005817/ci5034sup1.cif

Structure factors: contains datablocks I. DOI: 10.1107/S1600536810005817/ci5034Isup2.hkl

Additional supplementary materials:  crystallographic information; 3D view; checkCIF report

## Figures and Tables

**Fig. 1.**
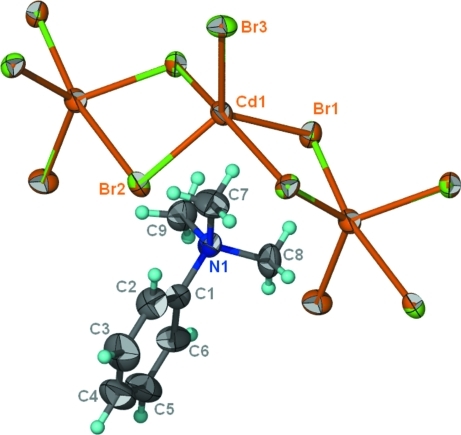
Displacement ellipsoid plot (Barbour, 2001) of a portion of polymeric C9H14N+ [CdBr2Cl]- at the 50% probability level. H are drawn as spheres of arbitrary radius. The disorder in the halogen sites not shown.

## supplementary crystallographic information

### Experimental

Cadmium chloride hemipentahydrate (0.45 g , 2 mmol) and trimethylphenylammonium tribromide (0.76 g, 2mmol) were heated in ethanol for 1 h. After filtering of the reaction mixture, colourless crystals were obtained upon slow evaporation of the yellow filtrate.

### Refinement

The aromatic ring was refined as a rigid hexagon (C—C = 1.39 Å). The N—C_methyl_ distances were restrained to 1.50 (1) Å. H atoms were placed at calculated positions (C–H = 0.93–0.96 Å) and were treated as riding on their parent atoms, with *U*(H) set to 1.2–1.5 times *U*_eq_(C).

Each of the three halogen sites are occupied by Cl or Br atoms. The total site occupancy of the Cl atoms refined to nearly 1 and that of Br atoms to nearly 2. Hence, the total site occupancy was fixed as 1.0 for Cl and 2.0 for Br atoms. The same U^ij^ parameters were used for Br and Cl atoms occupying the same site.

### Crystal data

**Table:**

(C_9_H_14_N)[CdBr_2_Cl]	*F*(000) = 840
*M**_r_* = 443.88	*D*_x_ = 2.106 Mg m^−^^3^
Monoclinic, *C**c*	Mo *K*α radiation, λ = 0.71073 Å
Hall symbol: C -2yc	Cell parameters from 5267 reflections
*a* = 12.9403 (2) Å	θ = 2.7–28.3°
*b* = 14.7059 (2) Å	µ = 7.43 mm^−^^1^
*c* = 7.3866 (1) Å	*T* = 293 K
β = 95.1590 (8)°	Block, colourless
*V* = 1399.97 (3) Å^3^	0.30 × 0.25 × 0.20 mm
*Z* = 4	

### Data collection

**Table:**

Bruker SMART APEX diffractometer	3068 independent reflections
Radiation source: fine-focus sealed tube	2966 reflections with *I* > 2σ(*I*)
graphite	*R*_int_ = 0.026
ω scans	θ_max_ = 27.5°, θ_min_ = 2.1°
Absorption correction: multi-scan (*SADABS*; Sheldrick, 1996)	*h* = −16→16
*T*_min_ = 0.378, *T*_max_ = 0.746	*k* = −19→19
6431 measured reflections	*l* = −9→9

### Refinement

**Table:**

Refinement on *F*^2^	Secondary atom site location: difference Fourier map
Least-squares matrix: full	Hydrogen site location: inferred from neighbouring sites
*R*[*F*^2^ > 2σ(*F*^2^)] = 0.027	H-atom parameters constrained
*wR*(*F*^2^) = 0.070	*w* = 1/[σ^2^(*F*_o_^2^) + (0.0296*P*)^2^ + 0.0436*P*] where *P* = (*F*_o_^2^ + 2*F*_c_^2^)/3
*S* = 1.06	(Δ/σ)_max_ = 0.001
3068 reflections	Δρ_max_ = 0.67 e Å^−^^3^
124 parameters	Δρ_min_ = −0.68 e Å^−^^3^
10 restraints	Absolute structure: Flack (1983), 1451 Friedel pairs
Primary atom site location: structure-invariant direct methods	Flack parameter: 0.021 (9)

### Fractional atomic coordinates and isotropic or equivalent isotropic displacement parameters (Å^2^)

**Table:**

	*x*	*y*	*z*	*U*_iso_*/*U*_eq_	Occ. (<1)
Cd1	0.50000 (2)	0.451513 (19)	0.50000 (3)	0.04131 (10)	
Br1	0.63269 (6)	0.51023 (6)	0.29653 (9)	0.04153 (19)	0.302 (2)
Br2	0.36460 (4)	0.55946 (3)	0.61951 (6)	0.04104 (14)	0.861 (2)
Br3	0.48809 (5)	0.28104 (3)	0.54560 (8)	0.05001 (16)	0.837 (2)
Cl1	0.63269 (6)	0.51023 (6)	0.29653 (9)	0.04153 (19)	0.698 (2)
Cl2	0.36460 (4)	0.55946 (3)	0.61951 (6)	0.04104 (14)	0.139 (2)
Cl3	0.48809 (5)	0.28104 (3)	0.54560 (8)	0.05001 (16)	0.163 (2)
N1	0.6446 (3)	0.8091 (3)	0.5732 (4)	0.0399 (8)	
C1	0.5525 (2)	0.8692 (2)	0.5708 (5)	0.0405 (9)	
C2	0.4530 (3)	0.8337 (2)	0.5711 (6)	0.0623 (14)	
H2	0.4432	0.7710	0.5716	0.075*	
C3	0.3682 (2)	0.8917 (3)	0.5705 (8)	0.089 (2)	
H3	0.3017	0.8679	0.5706	0.106*	
C4	0.3830 (3)	0.9854 (3)	0.5696 (8)	0.091 (3)	
H4	0.3262	1.0242	0.5692	0.109*	
C5	0.4825 (4)	1.02091 (19)	0.5694 (7)	0.082 (2)	
H5	0.4923	1.0835	0.5688	0.098*	
C6	0.5673 (3)	0.9628 (2)	0.5700 (6)	0.0594 (14)	
H6	0.6339	0.9866	0.5698	0.071*	
C7	0.6156 (6)	0.7104 (3)	0.5704 (10)	0.0697 (16)	
H7A	0.5714	0.6975	0.4620	0.104*	
H7B	0.5796	0.6966	0.6751	0.104*	
H7C	0.6773	0.6740	0.5722	0.104*	
C8	0.7023 (4)	0.8299 (5)	0.4097 (7)	0.0624 (14)	
H8A	0.6595	0.8148	0.3009	0.094*	
H8B	0.7650	0.7946	0.4153	0.094*	
H8C	0.7193	0.8934	0.4086	0.094*	
C9	0.7151 (4)	0.8261 (5)	0.7446 (7)	0.0623 (15)	
H9A	0.7481	0.8843	0.7365	0.093*	
H9B	0.7670	0.7794	0.7583	0.093*	
H9C	0.6749	0.8255	0.8476	0.093*	

### Atomic displacement parameters (Å^2^)

**Table:**

	*U*^11^	*U*^22^	*U*^33^	*U*^12^	*U*^13^	*U*^23^
Cd1	0.04678 (18)	0.03161 (15)	0.04784 (18)	0.00106 (13)	0.01690 (13)	−0.00100 (13)
Br1	0.0383 (4)	0.0526 (5)	0.0341 (3)	−0.0033 (3)	0.0054 (3)	0.0011 (3)
Br2	0.0381 (3)	0.0426 (3)	0.0431 (3)	0.00585 (18)	0.00755 (18)	−0.00786 (17)
Br3	0.0605 (3)	0.0296 (2)	0.0590 (3)	0.0028 (2)	0.0003 (2)	0.00449 (19)
Cl1	0.0383 (4)	0.0526 (5)	0.0341 (3)	−0.0033 (3)	0.0054 (3)	0.0011 (3)
Cl2	0.0381 (3)	0.0426 (3)	0.0431 (3)	0.00585 (18)	0.00755 (18)	−0.00786 (17)
Cl3	0.0605 (3)	0.0296 (2)	0.0590 (3)	0.0028 (2)	0.0003 (2)	0.00449 (19)
N1	0.0381 (19)	0.042 (2)	0.0400 (18)	−0.0041 (15)	0.0046 (14)	0.0012 (14)
C1	0.040 (2)	0.040 (2)	0.041 (2)	−0.0041 (17)	0.0006 (17)	−0.0010 (16)
C2	0.047 (3)	0.068 (4)	0.072 (4)	−0.014 (3)	0.005 (2)	0.005 (3)
C3	0.046 (3)	0.120 (7)	0.098 (5)	0.005 (4)	0.001 (3)	−0.006 (5)
C4	0.084 (5)	0.103 (6)	0.082 (5)	0.047 (5)	−0.013 (4)	−0.011 (4)
C5	0.091 (5)	0.064 (4)	0.089 (5)	0.025 (4)	−0.003 (4)	−0.008 (4)
C6	0.065 (4)	0.040 (3)	0.071 (3)	−0.004 (2)	−0.004 (3)	0.005 (2)
C7	0.082 (4)	0.038 (3)	0.090 (5)	−0.001 (3)	0.007 (3)	−0.002 (3)
C8	0.046 (3)	0.093 (5)	0.050 (3)	0.001 (3)	0.017 (2)	−0.001 (3)
C9	0.051 (3)	0.085 (4)	0.048 (3)	0.002 (3)	−0.008 (2)	0.001 (3)

### Geometric parameters (Å, °)

**Table:**

Cd1—Br1	2.5332 (8)	C3—H3	0.93
Cd1—Br3	2.5361 (6)	C4—C5	1.39
Cd1—Br2	2.5782 (5)	C4—H4	0.93
Cd1—Cl1^i^	2.7178 (8)	C5—C6	1.39
Cd1—Br1^i^	2.7178 (8)	C5—H5	0.93
Cd1—Br2^ii^	3.1795 (5)	C6—H6	0.93
Br1—Cd1^ii^	2.7178 (8)	C7—H7A	0.96
N1—C1	1.483 (4)	C7—H7B	0.96
N1—C7	1.499 (6)	C7—H7C	0.96
N1—C8	1.507 (5)	C8—H8A	0.96
N1—C9	1.513 (5)	C8—H8B	0.96
C1—C2	1.39	C8—H8C	0.96
C1—C6	1.39	C9—H9A	0.96
C2—C3	1.39	C9—H9B	0.96
C2—H2	0.93	C9—H9C	0.96
C3—C4	1.39		
			
Br1—Cd1—Br3	117.92 (3)	C4—C3—H3	120.0
Br1—Cd1—Br2	120.74 (3)	C2—C3—H3	120.0
Br3—Cd1—Br2	120.79 (2)	C3—C4—C5	120.0
Br1—Cd1—Cl1^i^	89.70 (2)	C3—C4—H4	120.0
Br3—Cd1—Cl1^i^	98.00 (2)	C5—C4—H4	120.0
Br2—Cd1—Cl1^i^	89.78 (2)	C6—C5—C4	120.0
Br1—Cd1—Br1^i^	89.70 (2)	C6—C5—H5	120.0
Br3—Cd1—Br1^i^	98.00 (2)	C4—C5—H5	120.0
Br2—Cd1—Br1^i^	89.78 (2)	C5—C6—C1	120.0
Cl1^i^—Cd1—Br1^i^	0.00 (4)	C5—C6—H6	120.0
Br1—Cd1—Br2^ii^	80.904 (19)	C1—C6—H6	120.0
Br3—Cd1—Br2^ii^	91.835 (17)	N1—C7—H7A	109.5
Br2—Cd1—Br2^ii^	89.796 (16)	N1—C7—H7B	109.5
Cl1^i^—Cd1—Br2^ii^	168.79 (2)	H7A—C7—H7B	109.5
Br1^i^—Cd1—Br2^ii^	168.79 (2)	N1—C7—H7C	109.5
Cd1—Br1—Cd1^ii^	97.81 (3)	H7A—C7—H7C	109.5
C1—N1—C7	112.1 (4)	H7B—C7—H7C	109.5
C1—N1—C8	109.0 (4)	N1—C8—H8A	109.5
C7—N1—C8	109.1 (5)	N1—C8—H8B	109.5
C1—N1—C9	109.5 (4)	H8A—C8—H8B	109.5
C7—N1—C9	107.6 (4)	N1—C8—H8C	109.5
C8—N1—C9	109.4 (4)	H8A—C8—H8C	109.5
C2—C1—C6	120.0	H8B—C8—H8C	109.5
C2—C1—N1	121.3 (3)	N1—C9—H9A	109.5
C6—C1—N1	118.7 (3)	N1—C9—H9B	109.5
C3—C2—C1	120.0	H9A—C9—H9B	109.5
C3—C2—H2	120.0	N1—C9—H9C	109.5
C1—C2—H2	120.0	H9A—C9—H9C	109.5
C4—C3—C2	120.0	H9B—C9—H9C	109.5
			
Br3—Cd1—Br1—Cd1^ii^	103.65 (3)	C9—N1—C1—C2	−117.8 (4)
Br2—Cd1—Br1—Cd1^ii^	−67.89 (3)	C7—N1—C1—C6	−179.0 (4)
Cl1^i^—Cd1—Br1—Cd1^ii^	−157.45 (4)	C8—N1—C1—C6	−58.1 (4)
Br1^i^—Cd1—Br1—Cd1^ii^	−157.45 (4)	C9—N1—C1—C6	61.6 (4)
Br2^ii^—Cd1—Br1—Cd1^ii^	16.407 (19)	N1—C1—C2—C3	179.4 (4)
C7—N1—C1—C2	1.6 (5)	N1—C1—C6—C5	−179.5 (3)
C8—N1—C1—C2	122.5 (4)		

Symmetry codes: (i) *x*, −*y*+1, *z*+1/2; (ii) *x*, −*y*+1, *z*−1/2.
